# The intra- and interobserver variability of PSMA-expression scores in patients with primary prostate cancer

**DOI:** 10.1186/s13550-024-01152-z

**Published:** 2024-10-24

**Authors:** Maarten L. Donswijk, Rosemarijn H. Ettema, Suzanne van der Gaag, Maurits Wondergem, Zing Cheung, Henk G. van der Poel, André N. Vis, Daniela E. Oprea-Lager

**Affiliations:** 1 Department of Nuclear Medicine, The Netherlands Cancer Institute - Antoni Van Leeuwenhoek, Plesmanlaan 121, 1066 CX Amsterdam, The Netherlands; 2 Department of Urology, Amsterdam University Medical Centre Amsterdam, Amsterdam, The Netherlands; 3 Prostate Cancer Network, Amsterdam, The Netherlands; 4 Department of Urology, St. Antonius Hospital, Nieuwegein, The Netherlands; 5 Department of Radiology and Nuclear Medicine, University Medical Centre Utrecht, Utrecht, The Netherlands; 6 Department of Radiology and Nuclear Medicine, Amsterdam University Medical Centre Amsterdam, Amsterdam, The Netherlands; 7 Department of Nuclear Medicine, Noordwest Ziekenhuisgroep, Alkmaar, The Netherlands; 8 Department of Urology, The Netherlands Cancer Institute - Antoni Van Leeuwenhoek, Amsterdam, The Netherlands; 9 Department of Radiology and Nuclear Medicine, Radboud University Medical Center, Nijmegen, The Netherlands

## Introduction

Currently, prostate-specific membrane antigen (PSMA) positron emission tomography (PET) is increasingly employed for staging of patients with intermediate- and high-risk primary prostate cancer, as well as in patients experiencing recurrence after treatment with curative intent [1, 2]. The immunohistochemical expression of PSMA in primary prostate cancer correlates to the Gleason Grade [3]. Similarly, most prostate tumours with high Gleason Grades and other poor prognostic parameters have higher PSMA-expression rates, as assessed on PSMA PET/CT [4]. The initial version of the Prostate Cancer Molecular Imaging Standardized Evaluation (PROMISE) framework introduced a 4-point PSMA-expression score to describe the PSMA-expression of primary tumours and metastases, aiming to standardize the interpretation and reporting of PSMA-targeted imaging studies [5]. This PSMA-expression scoring system has been retained in the second version of the PROMISE framework [6] and is endorsed by the EANM guidelines [7, 8]. The PSMA-expression score has been primarily proposed and used as a visual scale. However, limited information exists on the observer variability of the PSMA-expression score. Variations in interpretation among nuclear medicine readers and between different PSMA PET tracers may affect diagnostic interpretation and clinical decision-making regarding patient eligibility for PSMA-ligand directed radionuclide therapy [7–9].

This study aimed to determine the intra- and interobserver variability in the assessment of the PSMA-expression score of the primary prostate tumour in patients staged with PSMA PET/CT prior to radical prostatectomy. In addition, the observer variability of a consensus-based quantitative assessment of the PSMA-expression score was investigated and compared with the visually assessed PSMA-expression score.

## Material and methods

### Patients

This study was an extension of the observer agreement analysis conducted in a previous retrospective multicentre cohort study on the value of PSMA PET for local prostate tumour staging [10]. In this previous study, a random sample of 100 patients was selected from a cohort of 600 consecutive, predominantly intermediate and high-risk primary prostate cancer patients who underwent PSMA PET/CT before robot-assisted radical prostatectomy as per protocol.

### PSMA PET imaging

PSMA PET imaging was performed in NCI-AVL, Amsterdam UMC or in one of the referring external centres, according to local protocols on EARL accredited PET systems and in accordance with the EANM guidelines on prostate cancer imaging [8, 11]. The applied PSMA tracers included the ^18^F-labelled tracers: [^18^F]DCFPyL, [^18^F]PSMA-1007, [^18^F]-JK-PSMA-7, and the ^68^Ga-labelled tracer [^68^Ga]Ga-PSMA-11. Relevant imaging details have been described previously [10].

### Image analysis and assessment of PSMA-expression score

In the first analysis, four nuclear medicine physicians with ample experience in PSMA PET/CT reading (i.e., > 5 years of experience and > 2000 scans) had been instructed to visually assess the PSMA-expression score of the primary prostate tumour on a 4-point scale according to PROMISE V1 criteria [5]. PSMA-expression scores were reported as: 0, no expression (below blood pool); 1, low (equal to or above blood pool and lower than liver); 2, intermediate (equal to or above liver and lower than the parotid gland); 3, high (equal to or above parotid gland).

The second analysis was part of an extension study. The same four observers conducted a live consensus training meeting prior to assessing the PSMA-expression score of the primary prostate tumour in the same cohort of patients as in the first analysis, according to PROMISE V2 criteria [6]. The cut-off levels of the PROMISE V2 criteria slightly differ from the PROMISE V1. In PROMISE V2, the PSMA-expression scores are: 0, no expression (equal to or below blood pool); 1, low (above blood pool and lower than or equal to liver); 2, intermediate (above liver and lower than or equal to parotid gland); 3, high (above parotid gland). During the training, consensus was achieved on specific aspects of the PROMISE criteria, such as using the right parotid gland as reference organ, and using the spleen as reference organ in [^18^F]PSMA-1007 studies, while using the liver as reference organ for the other PET tracers. Furthermore, consensus was reached on a quantification-based method of PSMA-expression assessment of the primary prostate tumour. In this, a volume of interest (VOI) was drawn over the prostate, measuring the maximum standardized uptake value (SUV_max_) of the hottest region in the prostate. This SUV_max_ was compared with the SUV_max_ values obtained from a 3.0 cm diameter sphere VOI in the ascending thoracic aorta, a 4.0 cm diameter sphere VOI in the right liver lobe (or spleen in case of [^18^F]PSMA-1007), and a VOI over the hottest region in the right parotid gland. Based on this comparison, a PSMA-expression score of the primary prostate tumour was assigned according to PROMISE V2 criteria.

All analyses were performed using clinical DICOM imaging software [12, 13]. Observers were blinded from all clinical and pathological information, clinical reports of the scans, and results of other imaging. In the second analysis, the quantification-based scores were assessed after assigning the visual scores.

### Assessment of observer variability of PSMA-expression score

For the first visual analysis according to PROMISE V1 criteria, the intra-observer variability was determined (I) by requesting the four observers to re-analyse 25 of their earlier assessed cases (100 in total) at least 6 months after initial analysis, ensuring absence of recollection of the individual cases. The interobserver variability (II) was determined by dual observer analysis of 100 cases using varying pairs of observers. Interobserver variability was also determined for the visual (III) and quantitative (IV) analysis after consensus training and according to PROMISE V2 criteria. Observers were blinded from earlier results. Finally, the intra-observer agreement of the visual and quantitative analysis after consensus training and according to PROMISE V2 criteria was determined (V).

### Statistical analysis

Statistical analyses were performed using the Statistical Package for Social Sciences (SPSS, IBM; v29). Agreement was given as a percentage and kappa coefficients using Cohen’s weighted kappa with 95% CI. Differences in PSMA-expression scores distribution across analyses was tested using Friedman Repeated test. All statistical tests were two-tailed, and a value of *p* ≤ 0.05 was considered statistically significant.

## Results

Clinical and histopathological characteristics of the included 100 patients are provided in Table 1. Imaging characteristics and frequency of assigned PSMA-expression scores per analysis are presented in Table 2. A significant difference in overall distribution of PSMA-expression scores was seen across analyses (*p* = 0.03), with notably lower number of score 1 and higher numbers of score 3 in PROMISE V1 analysis compared with V2 analyses. Overall and tracer-specific SUV_max_ values of the primary prostate tumour and reference organs are presented in Table 3.

**Table 1:** Clinical and histopathological characteristics of included patients (*n* = 100)

Age (years), median (IQR)	68 (63–72)
Initial serum PSA level (ng/ml), median (IQR)	10 (6–18)
D’Amico risk classification	
Low risk	2
Intermediate risk	45
High risk	53
Pathological grade group in prostatectomy specimen	
ISUP 1	2
ISUP 2	25
ISUP 3	25
ISUP 4	9
ISUP 5	39

*PSA* Prostate-specific antigen, *ISUP* International Society of Urological Pathology, *IQR* Interquartile range

**Table 2:** Imaging characteristics of included patients (*n* = 100)

PSMA PET/CT tracer	*n*	Tracer dose (MBq, median (IQR))	Tracer biodistribution time (minutes, median (IQR))
[^68^Ga]Ga-PSMA-11	47	109 (93–146)	55 (46–60)
[^18^F]DCFPyL	39	232 (196–301)	68 (58–118)
[^18^F]PSMA-1007	11	294 (165–317)	93 (87–104)
[^18^F]-JK-PSMA-7	2	263 (251-*nc*)	104 (90–118)

PSMA-expression score of prostate tumour			
	PROMISE V1 (visual)^†^*	PROMISE V2 (visual)^‡^*	PROMISE V2 (quantitative)^‡^*
0	8 (3%)	1 (1%)	0
1	94 (31%)	79 (39%)	82 (41%)
2	113 (38%)	76 (38%)	73 (37%)
3	85 (28%)	44 (22%)	45 (23%)

^†^300 assessments per analysis

^‡^200 assessments per analysis

*Significant difference in overall distribution of PSMA-expression scores *p* = 0.03 (Friedman Repeated test)

*nc* Non-computable, *MBq* Megabecquerel, *IQR* Interquartile range, *PSMA* Prostate-specific membrane antigen

**Table 3:** Overall and tracer-specific^†^ SUVmax values of primary prostate tumour and reference organs in 100 patients undergoing pre-operative staging PSMA PET/CT in primary prostate cancer

	SUVmax, mean [IQR)			
	All tracers	[^68^ Ga]Ga-PSMA-11	[^18^F]DCFPyL	[^18^F]PSMA-1007
Prostate tumour	11.0 [6.2–17.5]	9.2 [6.0–15.0]	11.2 [4.9–25.8]	12.5 [10.6–17.5]
Bloodpool	2.2 [1.8–2.5]	2.2 [1.9–2.6]	1.9 [1.5–2.3]	2.2 [1.9–2.4]
Liver	8.4 [6.8–10.0]	8.2 [6.7–9.0]	7.5 [6.5–9.0]	13.8 [11.2–18.7]
Spleen	–	–	–	12.3 [10.0–16.5]*
Salivary gland	21.7 [17.0–25.4]	22.4 [17.6–25.4]	18.7 [14.9–22.1]	25.7 [22.4–28.9]

^†^No tracer-specific values given for [^18^F]-JK-PSMA-7 due to low number of cases (*n* = 2)

*In 6/14 [^18^F]PSMA-1007 scans, SUVmax of spleen exceeded SUVmax of liver

**Fig. 1:**
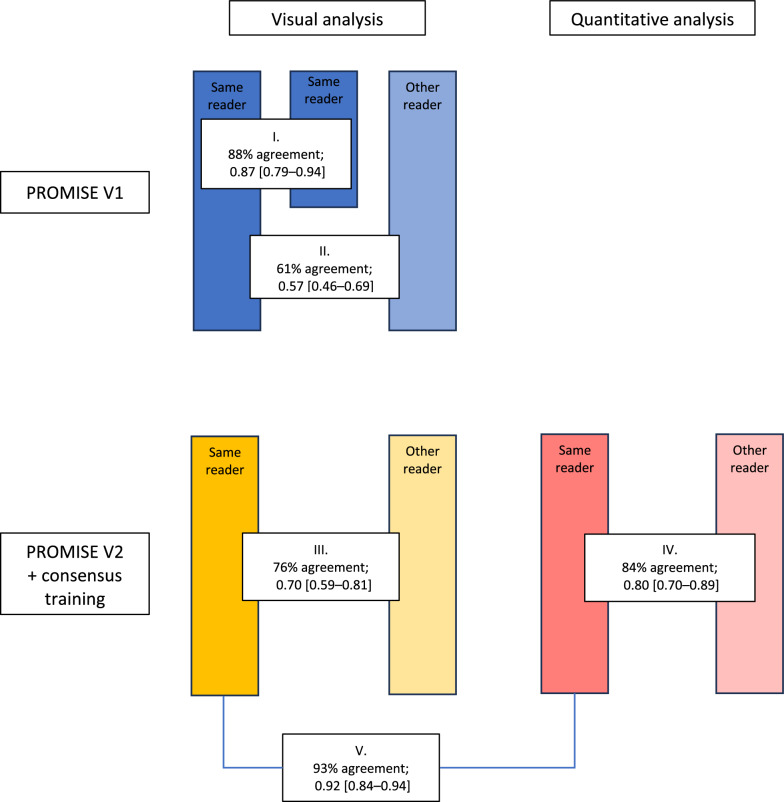
Graphical depiction of performed analyses and agreement of the PSMA-expression scores in 100 patients undergoing pre-operative staging PSMA PET/CT in primary prostate cancer. *κ* = kappa coefficients [95% confidence interval]

Results of the analyses are presented in Fig. 1. As conventionally classified [14], the intraobserver agreement (I) on the PSMA-expression score of the primary prostate tumour was nearly perfect, *κ* 0.87 [0.79–0.94]), while the interobserver agreement (II) was only moderate, *κ* 0.57 [0.46–0.69]). Following consensus training, the visual interobserver agreement (III) improved, *κ* 0.70 [0.59–0.81]. Using the quantitative analysis, the interobserver agreement (IV) further improved, *κ* 0.80 [0.70–0.89], significantly differing from the interobserver agreement observed in the first analysis (II). The intra-observer agreement of the visually assessed and quantification-based PSMA-expression scores (V) was nearly perfect, *κ* 0.92 [0.84–0.94].

For [^18^F]PSMA-1007 the intra-observer agreement was nearly perfect (*κ* 0.80 [0.43–1.00]) and in line with the other tracers; however, interobserver agreement was poor (*κ* 0.10 [-0.34–0.54]) and stayed significantly behind, even after consensus training (Table 4).

**Table 4:**
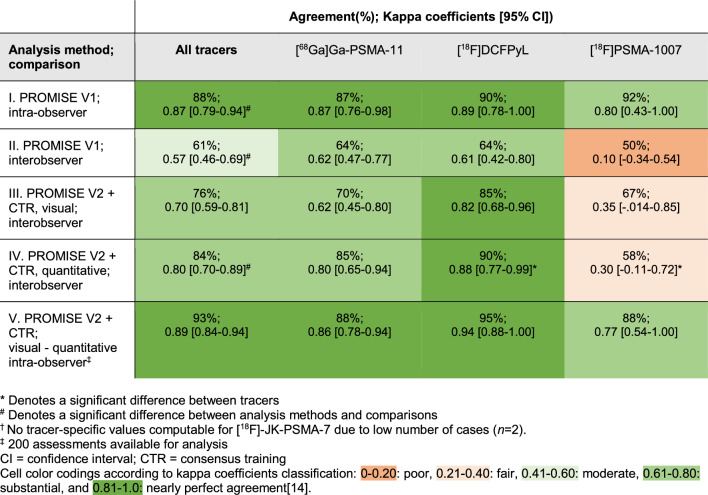
Overall and tracer-specific^†^ agreement of PSMA-expression scores in 100 patients undergoing pre-operative staging PSMA PET/CT in primary prostate cancer.

## Discussion

Present study, which investigated the intra- and interobserver variability of PSMA-expression scores of primary prostate tumours on PSMA PET/CT, showed that the intra-observer agreement was nearly perfect (*κ* 0.87), while the interobserver agreement was only moderate (*κ* 0.61).

Additionally, we demonstrated that following consensus training, the interobserver agreement rates improved compared with the agreement rates prior to consensus training. These findings emphasize the need for consensus training and clear interpretation criteria with minimal chance of differences in interpretation, even for experienced observers in tertiary referral centres. Moreover, the highest interobserver agreement rates were achieved through the quantification-based method using SUV_max_ of the primary tumour and reference organs (*κ* 0.80), thereby strongly advocating its use in daily practice. Quantification of PSMA-expression, such as by measurement of SUV_max_, is easily applied, and therefore should be encouraged.The intra-observer agreement of the visually assessed and quantification-based PSMA-expression score after consensus training was nearly perfect (*κ* 0.92), which confirms the high intra-observer concordance of the visual and quantification-based analysis methods.

Furthermore, we observed a significant difference in distribution of PSMA-expression scores comparing PROMISE V1 and V2 with the latter showing a shift to lower scores, likely a result of the different cut-off levels. Lastly, we observed remarkably lower interobserver agreement rates for [^18^F]PSMA-1007 compared with [^18^F]DCFPyL and [^68^Ga]Ga-PSMA-11. Due to the predominant biliary excretion of the [^18^F]PSMA-1007 tracer and hence physiologically high liver activity, consensus is that for [^18^F]PSMA-1007 the spleen with its generally more moderate activity, instead of liver, is the reference organ in determining the PSMA expression score [8]. Although the number of [^18^F]PSMA-1007 cases was limited, a considerable proportion (6/14) showed higher spleen activity than liver activity. This finding may have caused them to inadvertently use the liver instead of spleen as reference organ, and consequently may have played a role in the lower observer agreement for [^18^F]PSMA-1007. Furthermore, for [^18^F]PSMA-1007 median SUVmax of primary tumour was, compared to [^18^F]DCFPyL and [^68^Ga]Ga-PSMA-11, relatively close to median SUV_max_ liver and spleen (Table 3), which may have posed an additional challenge to consistently assess the PSMA expression score of the primary tumour related to these reference organs. Therefore, it is important to acknowledge the applied PROMISE criteria (V1 or V2) and the presence of differences in interobserver agreement of PSMA-expression scores when using different PSMA PET tracers [5, 6].

Derwael e.a. showed a substantial interobserver agreement of the PSMA-expression score of the primary tumour (79% agreement, Krippendorff’s alpha 0.69) in 43 primary prostate cancer patients undergoing [^68^Ga]Ga-PSMA-11 PET/CT [15]. However, agreement was only high in cases with PSMA-expression score 3, which constituted majority of cases (79%), whereas in our study expression score 3 accounted for only 28% of cases (visual analysis, PROMISE V1). Furthermore, our moderate initial interobserver agreement rates likely have been skewed by the poor agreement rates for [^18^F]PSMA-1007; in fact, agreement rates for [^18^F]DCFPyL and [^68^Ga]Ga-PSMA-11 were in line with this study. Also, Derwael e.a. found lower, albeit not statistically significant, interobserver agreement rates for the PSMA-expression score of lymph nodes compared to primary tumour and (distant) metastases. Our data only allowed us to assess the observer agreement of the PSMA-expression score of the primary tumour, not that of nodal or hematogenous metastases. However, our results did enable us to highlight a significant difference between intra- and interobserver agreement rates and the positive influence of consensus training and quantification on interobserver agreement rates. This, to our knowledge, has not been reported before.

The importance of robust interpretation criteria for PSMA-expression level on PSMA PET is clear considering its role in PSMA-ligand directed radionuclide therapy, for which “adequate” PSMA-expression levels of tumorous lesions are a prerequisite [9, 16]. However, valid questions remain about the definition of “adequate” PSMA-expression in tumorous lesions, whether defined as absolute (e.g. a SUV_max_ threshold) or as a minimum ratio relative to reference organs (liver, parotid). Although the latter method may be less scanner-dependent [9], our findings underline the reported confounder “reference-organ variability”, especially across different PSMA PET tracers. Furthermore, our findings demonstrate that interobserver variability of PSMA expression scores could be a non-negligible issue in therapy eligibility assessment. Future research may be aimed towards observer agreement assessment in the PSMA-ligand directed radionuclide therapy setting, as well as towards cross-validating the PSMA-expression scores across clinically used PSMA PET tracers.

Shortcomings of this study are the limited size of the cohort and the retrospective nature of certain data. Nevertheless, the results presented offer valuable insights into the robustness of the PSMA-expression score and ways for improvement of interobserver agreement rates.

## Conclusions

The intra-observer agreement of visually assessed PSMA-expression scores of primary prostate tumours according to PROMISE criteria was nearly perfect, while interobserver variability was only moderate and significantly differed across PSMA PET tracers. Consensus training and application of a quantification-based assessment using SUV_max_ significantly improved interobserver agreement rates.

## Data Availability

The datasets generated and analysed during the current study are not publicly available as these contain individual person’s data but are available from the corresponding author on reasonable request, after pseudonymization of the data and legal agreement.

## Abbreviations

CT: X-ray computed tomography
DICOM: Digital Imaging and Communications in Medicine
EANM: European Association of Nuclear Medicine
^18^F: Fluorine-18
[^18^F]-JK-PSMA-7: 2-Methoxy-^18^F-DCFPyL
[^18^F]DCFPyL: 2-(3-{1-Carboxy-5-[(6-[18F]fluoro-pyridine-3-carbonyl)-amino]-pentyl}-ureido)-pentanedioic acid
[18F]PSMA-1007: (2 ~ {S})-2-[[(2 ~ {S})-6-[[(2 ~ {S})-2-[[4-[[[(2 ~ {S})-2-[[(2 ~ {S})-2-[(6-fluoranylpyridin-3-yl)carbonylamino]-5-oxidanyl-5-oxidanylidene-pentanoyl]amino]-5-oxidanyl-5-oxidanylidene-pentanoyl]amino]methyl]phenyl]carbonylamino]-3-naphthalen-2-yl-propanoyl]amino]-1-oxidanyl-1-oxidanylidene-hexan-2-yl]carbamoylamino]pentanedioic acid
^68^Ga: Gallium-68
^68^Ga-PSMA-11: Glu-NH-CO–NH-Lys-(Ahx)-[^68^Ga]-HBED-CC
ISUP: International Society of Urologic Pathologists
PET: Positron emission tomography
PSA: Prostate specific antigen
PSMA: Prostate specific membrane antigen
PROMISE: Prostate Cancer Molecular Imaging Standardized Evaluation
SUV_max_: Maximum standardized uptake value
VOI: Volume of interest
